# Supplementary material for: Neuronal plasticity during motor rehabilitation training after spinal cord injury
Source: Commun Biol. 2026 Mar 9;9:561. doi: 10.1038/s42003-026-09793-7 (PMC13103311; doi:10.1038/s42003-026-09793-7)
Supplement: Supplementary file 1 — Supplementary Information [file 42003_2026_9793_MOESM1_ESM.pdf]

Supplementary Figures and Tables

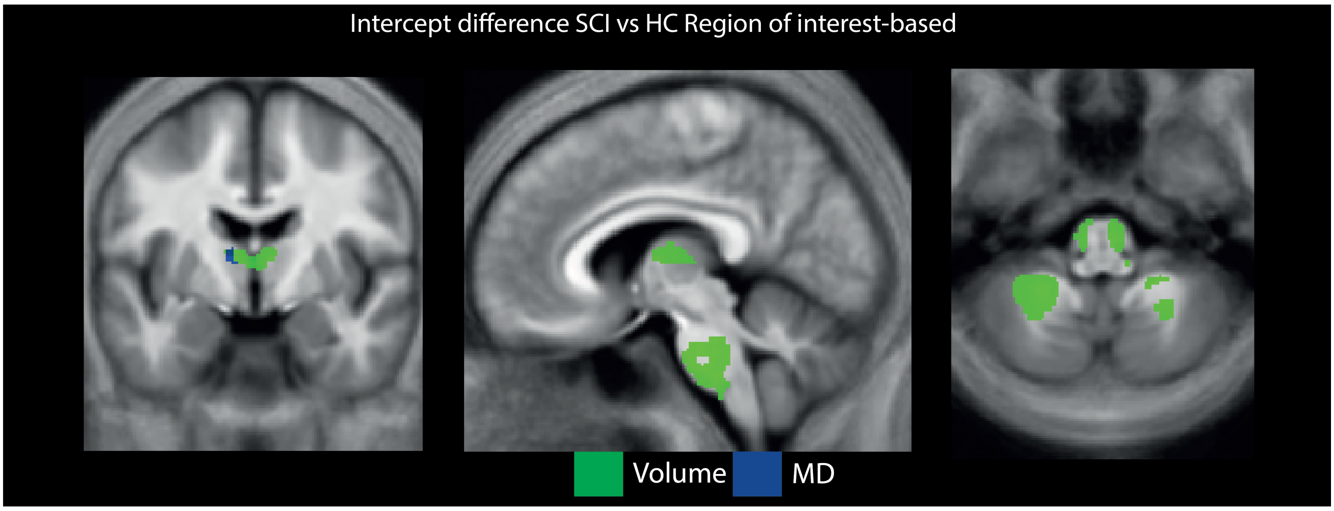

Supplementary Figure 1: Longitudinal SPM shows intercept differences in region of interest, white matter (WM) volume (green), and mean diffusivity (MD; blue) between healthy controls (HC) and spinal cord injured (SCI) patients. Showing significant WM volume decrease and MD increases in SCI patients. R=right, L=left.

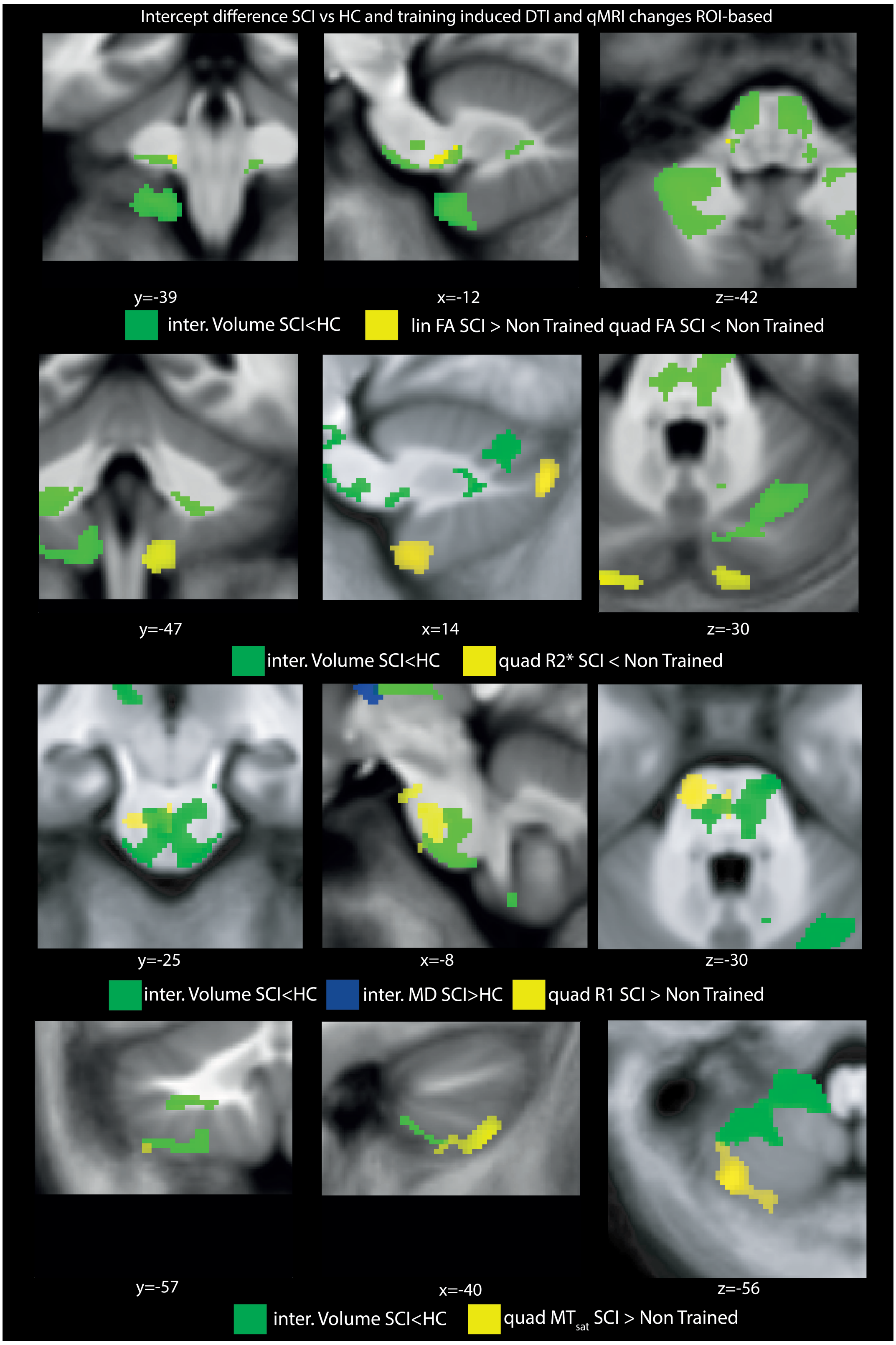

Supplementary Figure 2: Longitudinal changes in SPM plasticity among spinal cord injured patients (SCI), overlaid with intercept differences (SCI vs. healthy controls (HC)) from region of interest-based analysis. Grey matter (GM) volume and white matter (WM) volume differences are shown in green, and mean diffusivity (MD) in blue. The yellow overlay represents longitudinal SPM plasticity changes in the SCI group.

Training parameter	SCI Trainees Median (IQR)	SCI-ULlimb trainees Median (IQR)	SCI-LL limb trainee Median (IQR)	Healthy Trainees Median (IQR)	Healthy upper limb trainees Median (IQR)	Healthy lower limb trainees Median (IQR)	SCI-ULlimb trainees vs SCI-LL limb trainees p-value	SCI trainees vs Healthy trainees p-value	SCI-ULlimb trainees vs healthy upper limb trainees p-value	SCI-LL limb trainees vs healthy lower limb trainees p-value
baseline %CSR	47.8% (36.0 - 73.0%)	72.9% (51.0 - 76.1%)	40.0% (27.6 - 46.2%	78.0% (69.1 - 90.4%)	90.4% (87.3 - 90.8%)	66.9% (52.4 - 73.7%)	0.021	0.002	0.017	0.027
baseline RT	67.11 ms (62.98 - 72.58 ms)	66.26 ms (61.09 - 72.58 ms)	68.00 ms (63.24 - 72.00 ms)	58.24 ms (46.57 - 63.04 ms)	50.57 ms (40.57 - 60.93 ms)	60.92 ms (55.80 - 68.13 ms)	0.847	0.026	0.047	0.208
%CSR δ	35.9% (22.9 - 48.8%)	22.9% (19.4 - 35.4%)	46.4% (35.9 - 49.3%)	17.7% (11.2 - 23.2%)	11.2% (9.3 - 11.8%)	22.4% (21.9 - 29.0%)	0.054	0.001	0.002	0.007
RT δ	26.31 ms (14.73 - 40.21 ms)	39.50 ms (23.46 - 42.45 ms)	25.30 ms (10.52 - 27.4 ms)	25.77 ms (15.00 - 34.82 ms)	30.05 ms (13.18 - 34.82 ms)	25.38 ms (22.58 - 28.39 ms)	0.102	0.668	0.145	0.700
%CSR γ	0.10 (0.08 - 0.21)	0.09 (0.08 - 0.22)	0.11 (0.06 - 0.17)	0.17 (0.11 - 0.26)	0.14 (0.07 - 0.22)	0.17 (0.16 - 0.32)	0.700	0.070	0.895	0.043
RT γ	0.08 (0.05 - 0.13)	0.07 (0.05 - 0.13)	0.10 (0.06 - 0.13)	0.17 (0.10 - 0.28)	0.14 (0.11 - 0.27)	0.20 (0.10 - 0.28)	0.501	0.013	0.047	0.211

Supplementary Table 1: Characterization of performance improvements parameters of the patients and healthy control trainees, including percentage of correct stimulus response (%CSR), response time (RT) at baseline, improvement (δ), and improvement speed (γ). Note that behavioural data of the healthy trainees were published in Azzarito et al.[17].

ROI	MAP	contrast	p value (FWE-corrected)	cluster size	z-value	x[mm]	y[mm]	z[mm]
GM L cerebellum	VBM	intersept HC > SCI	<0.001	785	4.920	-15	-42	-57
GM L Thalamus	VBM	intersept HC > SCI	0.002	506	4.879	-4.5	-10.5	9
GM R cerebellum	VBM	intersept HC > SCI	0.001	643	4.639	22.5	-70.5	-28.5
GM R Thalamus	VBM	intersept HC > SCI	0.014	331	4.624	4.5	-9	10.5
WM L cerebellum	VBM	intersept HC > SCI	<0.001	1119	4.408	-33	-52.5	-43.5
WM R cerebellum	VBM	intersept HC > SCI	0.027	186	4.435	15	-66	-37.5
Corticospinal tract R	VBM	intersept HC > SCI	<0.001	987	4.177	7.5	-24	-42
Thalamus L	MD	intersept HC < SCI	<0.001	62	3.809	-10.5	-4.5	9
Corticospinal tract R	RD	intersept HC < SCI	0.013	21	3.968	34.5	-21	15

Supplementary Table 2: Longitudinal SPM shows intercept differences in region of interest, grey matter (GM) volume, white matter (WM) volume, mean diffusivity (MD), and radial diffusivity (RD) maps, between healthy controls (HC) and spinal cord injured (SCI) patients. R=right, L=left.

ROI	Map	Contrast	p-value (FEW-corrected)	Cluster size [voxels]	z-value	x [mm]	y [mm]	z [mm]	Fitted parameter SCI vs healthy trainees				Mean value in the cluster SCI trainees; 28 vs 84 days				Mean value in the cluster SCI vs healthy trainees at day 84		
									SCI	HC	p-value	BF_{01}	SCI at day 28	SCI at day 84	p-value	BF_{01}	HC at day 84	p-value	BF_{01}
GM L cerebellum	MT_{sat}	β_2 SCI trainees > Non-trainees	0.001	436	5.187	-39	-69	-54	267.918± 1034.335	-16.753± 64.388	0.274	1.818	1.892± 0.239 p.u.	1.906± 0.175 p.u.	0.620	3.495	1.989± 0.170 p.u.	0.162	1.401
GM R cerebellum	MT_{sat}	β_2 SCI trainees> Non-trainees	<0.001	1311	5.755	27	-79.5	-52.5	312.139± 1083.225	-10.738± 77.424	0.238	1.663	1.907± 0.217 p.u.	1.884± 0.181 p.u.	0.916	3.895	1.907± 0.116 p.u.	0.663	2.846
Corticospinal tract L at the level of the pons	R1	β_2 SCI trainees > Non-trainees	0.008	463	4.591	-12	-22.5	-33	55.418± 276.519	3.267± 31.266	0.451	2.404	0.989± 0.061 s^{-1}	0.994± 0.087 s^{-1}	0.863	3.862	1.031± 0.078 s^{-1}	0.199	1.577
R M1	VBM	β_1 SCI trainees > Non-trainees	0.021	195	6.927	27	-22.5	45	0.402± 1.084	-0.006± 0.092	0.142	1.168	0.065± 0.042 a.u.	0.056± 0.013 a.u.	0.249	2.123	0.052± 0.011 a.u.	0.431	2.394
Corticospinal tract L at the level of the corona radiata	VBM	β_1 SCI trainees > Non-trainees	0.008	98	>10	-24	-7.5	36	0.073± 0.325	-0.002± 0.007	0.356	2.122	0.705± 0.083 a.u.	0.714± 0.088 a.u.	0.200	1.834	0.771± 0.070 a.u.	0.044	0.573
Corticospinal tract L at the level of the corona radiata	VBM	β_1 SCI trainees > Non-trainees	0.024	72	>10	-25.5	10.5	21	0.159± 0.418	0.014± 0.033	0.172	1.341	0.688± 0.096 a.u.	0.691± 0.099 a.u.	0.451	3.012	0.761± 0.060 a.u.	0.018	0.279
Corticospinal tract L at the level of the corona radiata	VBM	β_2 SCI trainees < Non-trainees	<0.001	241	>10	-34.5	-9	27	-7.266± 23.540	0.228± 0.590	0.208	1.523	0.700± 0.083 a.u.	0.710± 0.087 a.u.	0.544	3.302	0.777± 0.076 a.u.	0.023	0.360
Corticospinal tract L at the level of the pons	VBM	β_2 SCI trainees < Non-trainees	0.002	130	>10	-4.5	-30	-33	-41.120± 162.910	0.163± 2.089	0.312	1.964	0.805± 0.101 a.u.	0.813± 0.092 a.u.	0.265	2.207	0.926± 0.111 a.u.	0.003	0.071
Corticospinal tract L at the level of the pons	VBM	β_2 SCI trainees < Non-trainees	0.001	145	>10	-19.5	-10.5	39	-7.575± 30.296	0.368± 0.694	0.296	1.904	0.720± 0.088 a.u.	0.728± 0.089 a.u.	0.452	3.015	0.782± 0.073 a.u.	0.060	0.717
WM L cerebellum	VBM	β_2 SCI trainees < Non-trainees	0.018	79	>10	-22.5	-51	-42	-18.658± 91.106	-0.093± 1.217	0.413	2.299	0.612± 0.110 a.u.	0.618± 0.103 a.u.	0.090	1.041	0.734± 0.084 a.u.	0.001	0.028
Corticospinal tract L at the level of the corona radiata	VBM	β_2 SCI trainees < Non-trainees	0.002	140	>10	-25.5	13.5	22.5	-6.247± 16.985	-0.104± 0.700	0.275	1.248	0.679± 0.096	0.691± 0.094 a.u.	0.213	1.913	0.756± 0.064 a.u.	0.024	0.349
Corticospinal tract R at the level of the pons	VBM	β_1 SCI trainees > Non-trainees	0.038	61	>10	4.5	-28.5	-37.5	0.731± 1.851	-0.003± 0.111	0.122	1.046	0.781± 0.117 a.u.	0.786± 0.117 a.u.	0.763	3.754	0.901± 0.113 a.u.	0.006	0.129
Corticospinal tract R at the level of the corona radiata	VBM	β_1 SCI trainees > Non-trainees	0.014	83	6.246	16.5	1.5	34.5	0.295± 1.194	0.009± 0.038	0.337	2.057	0.714± 0.083 a.u.	0.722± 0.088 a.u.	0.898	3.885	0.776± 0.081 a.u.	0.069	0.800
Corticospinal tract R at the level of the corona radiata	VBM	β_2 SCI trainees < Non-trainees	<0.001	1586	>10	25.5	-13.5	40.5	-9.642± 34.869	0.047± 0.297	0.269	1.796	0.702± 0.082 a.u.	0.713± 0.087 a.u.	0.369	2.698	0.777± 0.074 a.u.	0.025	0.380
WM R cerebellum	VBM	β_2 SCI trainees < Non-trainees	0.005	105	6.982	24	-46.5	-40.5	-20.956± 99.259	0.613± 1.691	0.384	2.210	0.638± 0.099 a.u.	0.637± 0.098 a.u.	0.512	3.211	0.738± 0.097 a.u.	0.004	0.102
Corticospinal tract L at the level of the corona radiata	FA	β_1 SCI trainees > Non-trainees	<0.001	71	>10	-34.5	-9	27	0.110± 0.366	0.001± 0.004	0.240	1.099	0.385± 0.046	0.387± 0.042	0.597	3.350	0.390± 0.036	0.821	2.247
Corticospinal tract L at the level of the corona radiata	FA	β_1 SCI trainees > Non-trainees	<0.001	77	>10	-21	-10.5	34.5	0.094± 0.238	0.002± 0.007	0.131	1.670	0.462± 0.047	0.470± 0.049	0.227	1.949	0.484± 0.044	0.377	3.013
Corticospinal tract L at the level of the corona radiata	FA	β_1 SCI trainees > Non-trainees	0.009	21	>10	-18	1.5	34.5	0.392± 1.535	0.015± 0.089	0.327	1.973	0.456± 0.061	0.470± 0.062	0.218	1.895	0.498± 0.012	0.152	0.503
Corticospinal tract L at the level of the corona radiata	FA	β_1 SCI trainees > Non-trainees	0.001	31	>10	-21	16.5	25.5	0.189± 0.684	-0.000± 0.003	0.269	1.798	0.410± 0.026	0.403± 0.027	0.156	1.509	0.411± 0.004	0.308	2.004
Corticospinal tract L at the level of the corona radiata	FA	β_1 SCI trainees > Non-trainees	<0.001	42	4.868	-31.5	-3	27	0.022± 0.089	0.000± 0.000	0.314	2.022	0.443± 0.037	0.440± 0.036	0.772	3.666	0.417± 0.006	0.038	1.331
WM L cerebellum	FA	β_2 SCI trainees < Non-trainees	<0.001	39	6.910	-13.5	-36	-42	-101.912± 383.704	2.543± 8.180	0.278	1.835	0.523± 0.035	0.528± 0.034	0.852	3.750	0.542± 0.005	0.156	1.338

Corticospinal tract R at the level of the corona radiata	FA	β_1 SCI trainees > Non-trainees	<0.001	182	>10	22.5	-12	33	0.100± 0.412	0.000± 0.000	0.332	2.039	0.445± 0.059	0.448± 0.057	0.564	3.269	0.464± 0.009	0.358	2.170
Corticospinal tract R at the level of the corona radiata	FA	β_2 SCI trainees < Non-trainees	0.016	20	>10	21	-13.5	42	-3.799± 15.663	0.000± 0.000	0.332	2.039	0.479± 0.073	0.483± 0.078	0.936	3.800	0.499± 0.011	0.491	2.529
Corticospinal tract L at the level of the corona radiata	AD	β_1 SCI trainees > Non-trainees	0.001	27	>10	-33	-10.5	27	(2.20± 7.54)·10^{-4}	(-0.00± 0.00)·10^{-4}	0.247	1.704	(9.54± 0.60)·10^{-4} mm²/s	(9.37± 0.43)·10^{-4} mm²/s.	0.212	1.861	(9.54± 0.16)·10^{-4} mm²/s	0.338	2.144
Corticospinal tract L at the level of the corona radiata	AD	β_1 SCI trainees > Non-trainees	<0.001	52	>10	-22.5	-15	34.5	(1.98± 7.63)·10^{-4}	(-0.01± 0.04)·10^{-4}	0.298	1.912	(10.26± 0.53)·10^{-4} mm²/s	(10.15± 0.54)·10^{-4} mm²/s	0.307	2.357	(10.32± 0.17)·10^{-4} mm²/s	0.341	2.136
Corticospinal tract R at the level of the corona radiata	AD	β_1 SCI trainees > Non-trainees	<0.001	182	>10	21	-19.5	46.5	(2.86± 11.80)·10^{-4}	(-0.00± 0.00)·10^{-4}	0.332	2.039	(10.32± 0.528)·10^{-4} mm²/s	(10.26± 0.48)·10^{-4} mm²/s	0.251	2.076	(10.31± 0.13)·10^{-4} mm²/s	0.800	2.998
Corticospinal tract R at the level of the corona radiata	AD	β_2 SCI trainees < Non-trainees	0.008	20	>10	21	-13.5	42	(-88.57± 365.17)·10^{-4}	(-0.00± 0.00)·10^{-4}	0.332	2.039	(10.83± 0.66)·10^{-4} mm²/s	(10.77± 0.61)·10^{-4} mm²/s	0.164	1.563	(10.80± 0.15)·10^{-4} mm²/s	0.896	3.054
Sensorimotor corteses L	MD	β_2 SCI trainees > Non-trainees	0.001	38	6.706	-31.5	-49.5	61.5	(879.94± 3288.14)·10^{-4}	(-12.07± 83.48)·10^{-4}	0.280	1.842	(9.40± 0.88)·10^{-4} mm²/s	(9.38± 1.16)·10^{-4} mm²/s	0.757	3.646	(8.83± 0.27)·10^{-4} mm²/s	0.170	1.445
Corticospinal tract L at the level of the corona radiata	RD	β_2 SCI trainees > Non-trainees	<0.001	54	>10	-21	-15	36	(59.83± 225.38)·10^{-4}	(0.70± 2.27)·10^{-4}	0.295	1.903	(5.15± 0.47)·10^{-4} mm²/s	(5.09± 1.00)·10^{-4} mm²/s	0.416	2.809	(4.94± 0.06)·10^{-4} mm²/s	0.279	1.882
Corticospinal tract L at the level of the corona radiata	RD	β_2 SCI trainees > Non-trainees	0.001	27	>10	-33	-9	27	(41.59± 133.38)·10^{-4}	(0.00± 0.00)·10^{-4}	0.217	1.566	5.02± 0.37)·10^{-4} mm²/s	4.96± 3.87)·10^{-4} mm²/s	0.407	2.777	(4.87± 0.06)·10^{-4} mm²/s	0.459	2.455
Corticospinal tract R at the level of the corona radiata	RD	β_1 SCI trainees < Non-trainees	0.002	24	>10	21	-10.5	42	(-1.05± 4.32)·10^{-4}	(0.00± 0.00)·10^{-4}	0.332	2.039	(4.76± 0.53)·10^{-4} mm²/s	(4.69± 0.56)·10^{-4} mm²/s	0.512	3.126	(4.54± 0.08)·10^{-4} mm²/s	0.336	2.096
Corticospinal tract R at the level of the corona radiata	RD	β_2 SCI trainees > Non-trainees	<0.001	190	>10	30	-12	36	(4.77± 0.19)·10^{-4}	(4.97± 0.23)·10^{-4}	0.010	2.039	(4.98± 0.43)·10^{-4} mm²/s	(4.77± 0.19)·10^{-4} mm²/s	0.321	3.126	(4.76± 0.06)·10^{-4} mm²/s	0.209	2.096

Supplementary Table 3: Longitudinal SPM shows linear (β_1) and quadratic (β_2) differences in grey matter (GM) volume, white matter (WM) volume, myelin-sensitive longitudinal relaxation rate (R1), magnetisation transfer rate (MTsat), fractional anisotropy (FA), mean diffusivity (MD), radial diffusivity (RD), and axial diffusivity (AD) maps between spinal cord injured (SCI) patients and trainees untrained healthy controls. Including the post-hoc analysis between spinal cord injured (SCI) trainees and healthy controls trainees (HC). R=right, L=left. p.u.= percent units. a.u. = arbitrary units.

ROI	MRI model parameter	Contrast	Direction of association	p value (FWE-corrected)	cluster size[voxels]	z-value	x [mm]	y [mm]	z [mm]
Cerebellum GM R	β_1 for MT_{sat}	RT improvement (δ)	negative	0.002	462	4.03	12	-73.5	-13.5
Sensorimotor cortex R	β_2 for VBM	RT improvement (δ)	positive	0.032	151	4.255	39	-36	33
CST at the level of the capsule internal R	β_2 for VBM	RT improvement speed (γ)	negative	0.008	101	5.805	18	-3	10.5
CST at the level of the corona radiata R	β_2 for VBM	%CSR plateauing (α)	negative	<0.001	245	3.735	22.5	-27	30
CST at the level of the corona radiata L	β_1 for FA	%CSR improvement speed (γ)	positive	<0.001	32	5.266	-27	-4.5	21
CST at the level of the corona radiata L	β_1 for FA	%CSR plateauing (α)	positive	<0.001	553	4.397	-15	-10.5	36
CST at the level of the corona radiata L	β_1 for FA	%CSR plateauing (α)	positive	<0.001	106	4.331	-30	-6	31.5
CST at the level of the corona radiata L	β_1 for FA	%CSR plateauing (α)	positive	0.001	27	3.486	-18	-31.5	33
CST at the level of the corona radiata L	β_1 for FA	%CSR plateauing (α)	positive	<0.001	53	3.287	-33	-28.5	33
Cerebellum WM L	β_2 for FA	%CSR plateauing (α)	negative	<0.001	210	5.436	-13.5	-33	-27
Cerebellum WM L	β_2 for FA	%CSR plateauing (α)	negative	<0.001	82	4.921	-36	-57	-40.5
Cerebellum WM L	β_2 for FA	%CSR plateauing (α)	negative	<0.001	39	4.725	-28.5	-43.5	-36
CST at the level of the corona radiata L	β_1 for FA	%CSR improvement (δ)	positive	<0.001	29	4.34	-30	-3	39
CST at the level of the corona radiata L	β_1 for FA	%CSR improvement (δ)	positive	<0.001	31	4.336	-31.5	-6	33
CST at the level of the corona radiata L	β_1 for FA	%CSR improvement (δ)	positive	<0.001	28	4.031	-13.5	-10.5	34.5
Cerebellum WM L	β_2 for FA	%CSR improvement (δ)	negative	<0.001	60	4.511	-19.5	-49.5	-33
CST at the level of the corona radiata R	β_1 for FA	%CSR plateauing (α)	positive	<0.001	136	5.145	25.5	1.5	39
CST at the level of the corona radiata R	β_1 for FA	%CSR plateauing (α)	positive	<0.001	504	4.346	25.5	-24	40.5
CST at the level of the corona radiata R	β_1 for FA	%CSR plateauing (α)	positive	0.002	27	4.286	15	12	31.5
CST at the level of the corona radiata R	β_1 for FA	%CSR plateauing (α)	positive	<0.001	114	4.274	25.5	19.5	30
CST at the level of the corona radiata R	β_1 for FA	%CSR plateauing (α)	positive	<0.001	71	3.514	33	4.5	22.5
CST at the level of the corona radiata R	β_2 for FA	%CSR plateauing (α)	negative	<0.001	47	5.876	30	-28.5	16.5
CST at the level of the corona radiata R	β_2 for FA	%CSR plateauing (α)	negative	<0.001	176	5.047	28.5	12	36
CST at the level of the corona radiata R	β_2 for FA	%CSR plateauing (α)	negative	0.003	29	4.904	19.5	-13.5	25.5
CST at the level of the crus cerebri R	β_2 for FA	%CSR plateauing (α)	negative	0.002	31	4.354	9	-9	-6
CST at the level of the capsula interna R	β_2 for FA	%CSR plateauing (α)	negative	<0.001	39	4.045	21	-15	-4.5
CST at the level of the corona radiata R	β_2 for FA	%CSR plateauing (α)	negative	0.003	29	3.429	28.5	-31.5	34.5
CST at the level of the corona radiata R	β_2 for FA	%CSR plateauing (α)	negative	<0.001	43	3.428	33	0	28.5
CST at the level of the corona radiata R	β_2 for FA	%CSR plateauing (α)	negative	0.019	20	3.149	25.5	0	21
CST at the level of the corona radiata R	β_1 for FA	%CSR improvement (δ)	positive	<0.001	34	5.366	15	-4.5	33
CST at the level of the corona radiata R	β_1 for FA	%CSR improvement (δ)	positive	<0.001	42	4.808	19.5	4.5	45
CST at the level of the corona radiata R	β_1 for FA	%CSR improvement (δ)	positive	<0.001	30	4.275	21	-21	40.5
CST at the level of the corona radiata L	β_1 for AD	%CSR improvement speed (γ)	positive	<0.001	31	5.262	-27	-6	22.5
CST at the level of the corona radiata L	β_1 for AD	%CSR plateauing (α)	positive	0.002	21	4.411	-15	-7.5	6
CST at the level of the corona radiata L	β_1 for AD	%CSR plateauing (α)	positive	<0.001	155	4.302	-30	-6	30
CST at the level of the corona radiata L	β_1 for AD	%CSR plateauing (α)	positive	<0.001	513	4.294	-30	-4.5	39

CST at the level of the corona radiata L	β_1 for AD	%CSR plateauing (α)	positive	<0.001	62	3.789	-13.5	12	34.5
CST at the level of the corona radiata L	β_1 for AD	%CSR plateauing (α)	positive	<0.001	24	3.664	-16.5	-30	31.5
CST at the level of the capsule interna L	β_1 for AD	%CSR plateauing (α)	positive	<0.001	20	3.647	-34.5	-22.5	16.5
CST at the level of the pons L	β_1 for AD	%CSR plateauing (α)	positive	<0.001	45	3.422	-6	-24	-45
CST at the level of the corona radiata L	β_1 for AD	%CSR improvement (δ)	positive	<0.001	29	4.342	-28.5	-1.5	37.5
CST at the level of the corona radiata L	β_1 for AD	%CSR improvement (δ)	positive	<0.001	28	4.293	-31.5	-6	33
CST at the level of the corona radiata L	β_1 for AD	%CSR improvement (δ)	positive	0.001	23	4.158	-13.5	-10.5	34.5
CST at the level of the crus cerebri R	β_2 for AD	RT improvement (δ)	positive	0.001	25	3.616	9	-25.5	-13.5
CST at the level of the corona radiata R	β_1 for AD	%CSR plateauing (α)	positive	<0.001	260	5.559	25.5	1.5	39
CST at the level of the capsule interna R	β_1 for AD	%CSR plateauing (α)	positive	<0.001	188	4.678	16.5	-7.5	15
CST at the level of the corona radiata R	β_1 for AD	%CSR plateauing (α)	positive	<0.001	490	4.351	21	-24	43.5
CST at the level of the crus cerebri R	β_1 for AD	%CSR plateauing (α)	positive	<0.001	29	4.322	9	-9	-13.5
CST at the level of the corona radiata R	β_1 for AD	%CSR plateauing (α)	positive	<0.001	29	4.14	15	12	31.5
CST at the level of the corona radiata R	β_1 for AD	%CSR plateauing (α)	positive	<0.001	72	3.497	33	1.5	25.5
CST at the level of the corona radiata R	β_2 for AD	%CSR plateauing (α)	negative	<0.001	159	5.195	27	16.5	28.5
CST at the level of the crus cerebri R	β_2 for AD	%CSR plateauing (α)	negative	<0.001	32	4.066	18	-13.5	-4.5
CST at the level of the pons R	β_2 for AD	%CSR plateauing (α)	negative	<0.001	238	4.061	3	-27	-45
CST at the level of the corona radiata R	β_2 for AD	%CSR plateauing (α)	negative	0.001	30	3.533	31.5	-25.5	37.5
CST at the level of the corona radiata R	β_2 for AD	%CSR plateauing (α)	negative	<0.001	41	3.331	27	-3	36
CST at the level of the corona radiata R	β_2 for AD	%CSR plateauing (α)	negative	0.01	20	3.1	25.5	0	21
CST at the level of the corona radiata R	β_1 for AD	%CSR improvement (δ)	positive	<0.001	39	4.917	13.5	-9	31.5
CST at the level of the corona radiata R	β_1 for AD	%CSR improvement (δ)	positive	<0.001	37	4.893	19.5	4.5	45
CST at the level of the corona radiata R	β_1 for AD	%CSR improvement (δ)	positive	<0.001	26	4.308	21	-22.5	40.5
Sensorimotor cortex L	β_2 for MD	RT improvement (δ)	negative	<0.001	55	3.814	-54	-19.5	55
Sensorimotor cortex L	β_2 for MD	%CSR plateauing (α)	positive	<0.001	156	5.518	-52.5	-18	51
Sensorimotor cortex L	β_2 for MD	%CSR plateauing (α)	positive	<0.001	45	4.744	-3	-25.5	69
Sensorimotor cortex L	β_2 for MD	%CSR plateauing (α)	positive	<0.001	171	4.601	-49.5	-7.5	43.5
Sensorimotor cortex L	β_2 for MD	%CSR plateauing (α)	positive	0.002	33	4.346	-51	-36	42
Sensorimotor cortex L	β_2 for MD	%CSR plateauing (α)	positive	<0.001	51	3.647	-39	-22.5	52.5
Sensorimotor cortex L	β_2 for MD	%CSR plateauing (α)	positive	<0.001	49	3.459	-18	-43.5	57
Sensorimotor cortex L	β_1 for MD	%CSR improvement (δ)	negative	0.028	20	4.602	-3	-25.5	73.5
CST at the level of the corona radiata L	β_2 for RD	%CSR plateauing (α)	positive	<0.001	170	3.97	-31.5	-7.5	30
CST at the level of the corona radiata L	β_2 for RD	%CSR plateauing (α)	positive	<0.001	147	3.547	-22.5	12	27
CST at the level of the corona radiata L	β_2 for RD	%CSR plateauing (α)	positive	<0.001	48	3.323	-36	-6	25.5
CST at the level of the corona radiata R	β_1 for RD	%CSR plateauing (α)	negative	<0.001	201	4.736	27	15	28.5
CST at the level of the corona radiata R	β_2 for RD	%CSR plateauing (α)	positive	0.003	25	5.356	19.5	-25.5	42
CST at the level of the corona radiata R	β_2 for RD	%CSR plateauing (α)	positive	0.008	21	5.187	15	10.5	37.5
CST at the level of the corona radiata R	β_2 for RD	%CSR plateauing (α)	positive	<0.001	143	4.562	27	16.5	21
CST at the level of the corona radiata R	β_2 for RD	%CSR plateauing (α)	positive	<0.001	73	4.203	13.5	-1.5	34.5
CST at the level of the corona radiata R	β_2 for RD	%CSR plateauing (α)	positive	<0.001	446	4.152	24	-21	37.5
CST at the level of the corona radiata R	β_2 for RD	%CSR improvement (δ)	positive	<0.001	27	3.992	19.5	-28.5	30

Supplementary Table 4: Correlations between the linear (β_1) and quadratic (β_2) components of the quadratic model describing the temporal evolution of MRI readouts and the performance improvement. MRI readouts include magnetization transfer rate (MTsat), volume changes (VBM), fractional anisotropy (FA), mean diffusivity (MD), radial diffusivity (RD), axial diffusivity (AD) maps and response time (RT) and percentual correct stimuli response (CSR%). For each combination of MRI readout, model parameter (β_1 or β_2), and performance improvement, only the largest cluster within a distinct brain region of interest (ROI) is shown. R = right, L = left

ROI	Map	contrast	p value (FWE-corrected)	cluster size [voxels]	z-value	x [mm]	y [mm]	z [mm]
Sensorimotor cortices L	MT_{sat}	β_2 SCI trainees Upper<Lower	0.029	219	4.304	-9	-46.5	76.5
Thalamus L	MT_{sat}	β_1 SCI trainees Upper>Lower	0.005	346	4.111	4.5	-15	10.5
Corticospinal tract at the level of the pons L	MT_{sat}	β_1 SCI trainees Upper>Lower	0.001	365	4.583	-4.5	-30	-33
Cerebellum WM L	MT_{sat}	β_1 SCI trainees Upper>Lower	0.006	248	4.482	-21	-49.5	-39
Corticospinal tract at the level of the pons R	MT_{sat}	β_1 SCI trainees Upper>Lower	<0.001	685	4.518	12	-33	-33
Corticospinal tract at the level of the pons L	R1	β_2 SCI trainees Upper<Lower	0.044	219	3.994	-1.5	-21	-27
Cerebellum WM R	R1	β_2 SCI trainees Upper>Lower	0.029	303	3.558	28.5	-55.5	-40.5
Corticospinal tract at the level of the corona radiata L	VBM	β_1 SCI trainees Upper<Lower	0.006	105	>10	-27	12	21
Corticospinal tract at the level of the corona radiata L	VBM	β_1 SCI trainees Upper<Lower	<0.001	234	>10	-24	-7.5	36
Corticospinal tract at the level of the pons L	VBM	β_1 SCI trainees Upper<Lower	0.034	65	6.142	-12	-40.5	-40.5
Corticospinal tract at the level of the corona radiata L	VBM	β_1 SCI trainees Upper>Lower	<0.001	195	>10	-33	-10.5	27
Corticospinal tract at the level of the pons L	VBM	β_1 SCI trainees Upper>Lower	<0.001	203	>10	-4.5	-30	-33
Corticospinal tract at the level of the corona radiata L	VBM	β_1 SCI trainees Upper>Lower	0.001	154	>10	-19.5	-10.5	39
Cerebellum WM L	VBM	β_1 SCI trainees Upper>Lower	<0.001	210	>10	-22.5	-51	-40.5
Corticospinal tract at the level of the corona radiata L	VBM	β_1 SCI trainees Upper>Lower	0.039	62	>10	-22.5	-28.5	46.5
Corticospinal tract at the level of the corona radiata L	VBM	β_2 SCI trainees Upper<Lower	<0.001	252	>10	-24	-7.5	36
Corticospinal tract at the level of the corona radiata L	VBM	β_2 SCI trainees Upper>Lower	<0.001	210	>10	-33	-10.5	27
Corticospinal tract at the level of the pons L	VBM	β_2 SCI trainees Upper>Lower	0.001	162	>10	-4.5	-30	-33
Corticospinal tract at the level of the corona radiata L	VBM	β_2 SCI trainees Upper>Lower	0.001	146	>10	-19.5	-10.5	39
Cerebellum WM L	VBM	β_2 SCI trainees Upper>Lower	0.029	68	>10	-22.5	-51	-40.5
Corticospinal tract at the level of the corona radiata L	VBM	β_2 SCI trainees Upper>Lower	0.035	64	>10	-22.5	-28.5	46.5
Corticospinal tract at the level of the corona radiata L	VBM	β_2 SCI trainees Upper>Lower	0.003	126	>10	-25.5	13.5	22.5
Corticospinal tract at the level of the corona radiata R	VBM	β_1 SCI trainees Upper<Lower	0.036	62	7.005	16.5	1.5	34.5
Corticospinal tract at the level of the corona radiata R	VBM	β_1 SCI trainees Upper>Lower	<0.001	1470	>10	25.5	-13.5	40.5
Cerebellum WM R	VBM	β_1 SCI trainees Upper>Lower	<0.001	197	>10	22.5	-46.5	-40.5
Corticospinal tract at the level of the corona radiata R	VBM	β_2 SCI trainees Upper<Lower	0.003	121	6.967	16.5	3	34.5
Corticospinal tract at the level of the pons R	VBM	β_2 SCI trainees Upper<Lower	0.036	62	4.371	6	-27	-15
Corticospinal tract at the level of the corona radiata R	VBM	β_2 SCI trainees Upper>Lower	<0.001	1430	>10	25.5	-13.5	40.5
Cerebellum WM R	VBM	β_2 SCI trainees Upper>Lower	0.008	95	6.918	24	-46.5	-40.5
Corticospinal tract at the level of the corona radiata L	FA	β_1 SCI trainees Upper<Lower	<0.001	109	>10	-33	-9	27
Corticospinal tract at the level of the corona radiata L	FA	β_1 SCI trainees Upper<Lower	<0.001	62	>10	-22.5	-15	34.5
Corticospinal tract at the level of the corona radiata L	FA	β_1 SCI trainees Upper<Lower	0.0024	27	>10	-18	1.5	34.5
Corticospinal tract at the level of the corona radiata L	FA	β_1 SCI trainees Upper<Lower	0.001	31	>10	-21	16.5	25.5
Corticospinal tract at the level of the pons L	FA	β_1 SCI trainees Upper<Lower	0.003	26	6.914	-4.5	-27	-30
Cerebellar peduncle L	FA	β_1 SCI trainees Upper>Lower	0.011	20	6.623	-12	-42	-37.5
Corticospinal tract at the level of the corona radiata R	FA	β_1 SCI trainees Upper<Lower	<0.001	182	>10	22.5	-13.5	33
Corticospinal tract at the level of the corona radiata R	FA	β_1 SCI trainees Upper>Lower	0.002	30	>10	25.5	-12	43.5
Corticospinal tract at the level of the corona radiata L	AD	β_1 SCI trainees Upper<Lower	0.002	25	>10	-36	-9	27
Corticospinal tract at the level of the corona radiata R	AD	β_1 SCI trainees Upper<Lower	<0.001	52	>10	-22.5	-15	34.5

Corticospinal tract at the level of the corona radiata R	AD	β_1 SCI trainees Upper<Lower	<0.001	182	>10	22.5	-18	46.5
Corticospinal tract at the level of the corona radiata R	AD	β_1 SCI trainees Upper>Lower	<0.001	31	>10	21	-10.5	42
Sensorimotorcorteses L	MD	β_1 SCI trainees Upper<Lower	<0.001	45	6.134	-31.5	-46.5	60
Corticospinal tract at the level of the corona radiata L	RD	β_2 SCI trainees Upper<Lower	<0.001	54	>10	-21	-15	36
Corticospinal tract at the level of the corona radiata L	RD	β_2 SCI trainees Upper<Lower	0.001	25	>10	-33	-9	27
Corticospinal tract at the level of the corona radiata R	RD	β_2 SCI trainees Upper<Lower	<0.001	191	>10	30	-12	36

Supplementary Table 5: Longitudinal statistical parametric mapping (SPM) analysis showing differences in the linear (β_1) and quadratic (β_2) time dependence for longitudinal rate (R1) and magnetisation transfer rate (MTsat), and transverse relaxation rate (R2*), fractional anisotropy (FA), mean diffusivity (MD), radial diffusivity (RD), and axial diffusivity (AD) maps between all spinal cord injured (SCI) upper limb trainees and all lower limb trainees. R=right, L=left.

ROI	Map	Contrast	p value (FWE-corrected)	cluster size [voxels]	z-value	x [mm]	y [mm]	z [mm]
WM L	FA	β_1 SCI-UL> CON-UL	0.002	29	>10	-25.5	-9	22.5
WM L	FA	β_1 SCI-UL> CON-UL	0.007	22	>10	-34.5	-6	33
WM L	FA	β_1 SCI-UL< CON-UL	0.001	34	5.996	-4.5	-28.5	-31.5
WM L	FA	β_2 SCI-UL< CON-UL	0.002	28	4.823	-27	-19.5	-1.5
WM L	FA	β_1 SCI-LL > CON-LL	<0.001	68	>10	-33	-9	27
WM L	FA	β_1 SCI-LL > CON-LL	<0.001	62	>10	-21	-10.5	34.5
WM L	FA	β_1 SCI-LL > CON-LL	0.001	31	>10	-21	16.5	25.5
WM L	FA	β_1 SCI-LL > CON-LL	<0.001	43	5.062	-30	-4.5	27
WM L	FA	β_1 SCI-LL < CON-LL	0.007	22	6.281	-12	-42	-37.5
WM L	FA	β_2 SCI-LL > CON-LL	<0.001	69	>10	-33	-10.5	27
WM L	FA	β_2 SCI-LL > CON-LL	<0.001	56	>10	-22.5	-15	34.5
WM L	FA	β_2 SCI-LL > CON-LL	<0.001	42	>10	-24	12	19.5
WM L	FA	β_2 SCI-LL > CON-LL	<0.001	43	5.211	-28.5	-3	27
WM L	FA	β_2 SCI-LL < CON-LL	0.002	29	6.545	-13.5	-36	-42
WM R	FA	β_1 SCI-UL> CON-UL	<0.001	98	>10	21	-10.5	33
WM R	FA	β_1 SCI-UL> CON-UL	0.016	20	>10	21	-13.5	42
WM R	FA	β_1SCI-UL> CON-UL	<0.001	40	>10	24	-24	43.5
WM R	FA	β_1 SCI-UL< CON-UL	<0.001	275	>10	30	-12	36
WM R	FA	β_1 SCI-UL< CON-UL	<0.001	48	6.207	1.5	-27	-30
WM R	FA	β_1 SCI-UL< CON-UL	0.002	31	5.719	28.5	7.5	37.5
WM R	FA	β_2 SCI-UL> CON-UL	<0.001	196	>10	30	-12	36
WM R	FA	β_2 SCI-UL> CON-UL	0.002	30	>10	27	6	31.5
WM R	FA	β_2 SCI-UL< CON-UL	<0.001	72	>10	21	-10.5	33
WM R	FA	β_2 SCI-UL< CON-UL	0.006	25	>10	24	-25.5	45
WM R	FA	β_1 SCI-LL > CON-LL	<0.001	182	>10	22.5	-10.5	34.5
WM R	FA	β_1 SCI-LL < CON-LL	0.002	30	>10	25.5	-12	43.5
WM R	FA	β_2 SCI-LL > CON-LL	<0.001	184	>10	30	-12	36
WM R	FA	β_2 SCI-LL < CON-LL	0.016	20	>10	21	-13.5	42
WM L	AD	β_1 SCI-UL> CON-UL	<0.001	55	>10	-21	-12	36
WM L	AD	β_1 SCI-UL> CON-UL	<0.001	37	>10	-34.5	-9	27
WM L	AD	β_1 SCI-UL> CON-UL	0.001	28	>10	-25.5	-6	21
WM L	AD	β_1 SCI-UL< CON-UL	<0.001	33	6.976	-7.5	-42	-37.5
WM L	AD	β_1 SCI-UL< CON-UL	<0.001	35	6.050	-4.5	-28.5	-31.5
WM L	AD	β_2 SCI-UL> CON-UL	0.006	20	>10	-24	-1.5	21
WM L	AD	β_2 SCI-UL< CON-UL	<0.001	41	>10	-21	-12	36
WM L	AD	β_2 SCI-UL< CON-UL	0.002	24	>10	-21	-16.5	46.5
WM L	AD	β_2 SCI-UL< CON-UL	<0.001	36	5.040	-25.5	-18	3
WM L	AD	β_1 SCI-LL > CON-LL	0.001	27	>10	-33	-10.5	27
WM L	AD	β_1 SCI-LL > CON-LL	<0.001	52	>10	-22.5	-15	34.5
WM L	AD	β_2 SCI-LL > CON-LL	<0.001	52	>10	-21	-12	36

WM L	AD	β_2 SCI-LL > CON-LL	0.002	25	>10	-33	-9	27
WM R	AD	β_1 SCI-UL> CON-UL	<0.001	305	>10	30	-12	36
WM R	AD	β_1 SCI-UL< CON-UL	<0.001	101	>10	19.5	-12	36
WM R	AD	β_1 SCI-UL< CON-UL	0.008	20	>10	21	-13.5	42
WM R	AD	β_1 SCI-UL< CON-UL	<0.001	45	>10	27	1.5	33
WM R	AD	β_1 SCI-UL< CON-UL	<0.001	52	5.772	4.5	-25.5	-33
WM R	AD	β_2 SCI-UL> CON-UL	<0.001	198	>10	30	-12	36
WM R	AD	β_2 SCI-UL< CON-UL	<0.001	77	>10	19.5	-12	36
WM R	AD	β_2 SCI-UL< CON-UL	0.003	24	>10	21	-24	40.5
WM R	AD	β_1 SCI-LL > CON-LL	<0.001	182	>10	22.5	-18	46.5
WM R	AD	β_1 SCI-LL < CON-LL	0.001	31	>10	21	-10.5	42
WM R	AD	β_2 SCI-LL > CON-LL	<0.001	185	>10	30	-12	36
WM R	AD	β_2 SCI-LL < CON-LL	0.008	20	>10	21	-13.5	42
GM L	MD	β_1 SCI-UL> CON-UL	0.002	36	>10	-45	-84	-28.5
GM L	MD	β_1 SCI-UL> CON-UL	<0.001	242	>10	-7.5	-93	-34.5
GM L	MD	β_1 SCI-UL> CON-UL	0.004	32	6.390	-19.5	-42	81
GM L	MD	β_1 SCI-UL> CON-UL	0.001	40	4.227	-21	-73.5	-46.5
GM L	MD	β_1 SCI-UL< CON-UL	<0.001	98	6.232	-52.5	-70.5	-36
GM L	MD	β_1 SCI-UL< CON-UL	<0.001	51	5.027	-1.5	-31.5	79.5
GM L	MD	β_1 SCI-UL< CON-UL	<0.001	98	3.809	-25.5	-90	-40.5
GM L	MD	β_1 SCI-UL< CON-UL	0.010	27	3.801	-19.5	-81	-57
GM L	MD	β_1 SCI-UL< CON-UL	0.016	25	3.801	0	-85.5	-49.5
GM L	MD	β_2 SCI-UL> CON-UL	<0.001	37	>10	-45	-84	-28.5
GM L	MD	β_2 SCI-UL> CON-UL	0.020	24	>10	-54	-15	60
GM L	MD	β_2 SCI-UL> CON-UL	0.002	36	7.458	-21	-43.5	81
GM L	MD	β_2 SCI-UL> CON-UL	0.025	23	5.822	-22.5	-72	-48
GM L	MD	β_2 SCI-UL> CON-UL	0.039	21	3.697	-13.5	-42	-4.5
GM L	MD	β_2 SCI-UL> CON-UL	0.001	38	3.371	-16.5	-31.5	-12
GM L	MD	β_2 SCI-UL< CON-UL	<0.001	155	6.318	-30	-43.5	51
GM L	MD	β_2 SCI-UL< CON-UL	0.001	38	5.791	0	-88.5	-36
GM L	MD	β_2 SCI-UL< CON-UL	<0.001	102	4.777	-25.5	-88.5	-43.5
GM L	MD	β_2 SCI-UL< CON-UL	0.010	27	4.763	-19.5	-82.5	-57
GM L	MD	β_2 SCI-UL< CON-UL	0.008	28	4.763	0	-84	-49.5
GM L	MD	β_2 SCI-UL< CON-UL	<0.001	44	3.502	-54	-64.5	-48
GM L	MD	β_1 SCI-LL > CON-LL	<0.001	61	>10	-7.5	-93	-39
GM L	MD	β_1 SCI-LL > CON-LL	0.001	42	6.173	-31.5	-46.5	60
GM L	MD	β_1 SCI-LL < CON-LL	<0.001	218	>10	-10.5	-94.5	-30
GM L	MD	β_1 SCI-LL < CON-LL	0.002	36	>10	0	-91.5	-36
GM L	MD	β_2 SCI-LL > CON-LL	<0.001	79	>10	-4.5	-90	-31.5
GM L	MD	β_2 SCI-LL > CON-LL	0.001	39	6.872	-31.5	-49.5	61.5
GM L	MD	β_2 SCI-LL < CON-LL	<0.001	206	>10	-15	-94.5	-36
GM L	MD	β_2 SCI-LL < CON-LL	0.002	35	>10	0	-91.5	-36

GM R	MD	β_1 SCI-UL> CON-UL	0.013	26	6.390	27	-94.5	-33
GM R	MD	β_1 SCI-UL> CON-UL	<0.001	50	4.315	15	-78	-25.5
GM R	MD	β_1 SCI-UL< CON-UL	<0.001	74	>10	9	-73.5	-55.5
GM R	MD	β_1 SCI-UL< CON-UL	<0.001	49	5.735	3	-33	79.5
GM R	MD	β_1 SCI-UL< CON-UL	<0.001	222	5.287	3	-90	-34.5
GM R	MD	β_2 SCI-UL> CON-UL	0.007	29	7.416	27	-94.5	-36
GM R	MD	β_2 SCI-UL> CON-UL	0.020	24	7.416	64.5	-16.5	45
GM R	MD	β_2 SCI-UL> CON-UL	<0.001	53	3.487	18	-67.5	-27
GM R	MD	β_2 SCI-UL< CON-UL	<0.001	232	6.208	6	-91.5	-34.5
GM R	MD	β_2 SCI-UL< CON-UL	0.016	25	5.278	12	-21	-10.5
GM R	MD	β_2 SCI-UL< CON-UL	0.001	38	4.611	36	-24	48
GM R	MD	β_1 SCI-LL > CON-LL	<0.001	171	>10	22.5	-75	-60
GM R	MD	β_1 SCI-LL > CON-LL	0.020	24	>10	13.5	-87	-49.5
GM R	MD	β_1 SCI-LL > CON-LL	0.016	25	>10	67.5	-18	36
GM R	MD	β_1 SCI-LL > CON-LL	<0.001	71	3.171	19.5	-12	-40.5
GM R	MD	β_1 SCI-LL > CON-LL	0.003	33	3.171	34.5	-43.5	73.5
GM R	MD	β_1 SCI-LL < CON-LL	<0.001	45	>10	3	-91.5	-37.5
GM R	MD	β_2 SCI-LL > CON-LL	<0.001	180	>10	21	-75	-63
GM R	MD	β_2 SCI-LL > CON-LL	0.020	24	>10	16.5	-91.5	-45
GM R	MD	β_2 SCI-LL > CON-LL	0.016	25	>10	67.5	-18	36
GM R	MD	β_2 SCI-LL > CON-LL	<0.001	71	3.297	22.5	-1.5	-48
GM R	MD	β_2 SCI-LL > CON-LL	0.003	33	3.297	37.5	-40.5	70.5
GM R	MD	β_2 SCI-LL < CON-LL	<0.001	44	>10	1.5	-90	-39
GM L	MT	β_1 SCI-LL > CON-LL	0.011	282	4.716	-37.5	-66	-55.5
GM L	MT	β_2 SCI-LL > CON-LL	<0.001	833	5.132	-36	-67.5	-55.5
GM R	MT	β_1 SCI-LL > CON-LL	<0.001	1023	5.094	27	-79.5	-52.5
GM R	MT	β_1 SCI-LL > CON-LL	0.001	503	4.591	55.5	-69	-48
GM R	MT	β_1 SCI-LL < CON-LL	0.002	413	4.385	31.5	-9	-22.5
GM R	MT	β_2 SCI-LL > CON-LL	<0.001	3066	6.278	25.5	-81	-52.5
GM R	MT	β_2 SCI-LL < CON-LL	0.007	325	3.997	22.5	-13.5	-21
WM L	MT	β_1 SCI-LL < CON-LL	0.001	388	4.654	-4.5	-31.5	-33
WM R	MT	β_1 SCI-LL < CON-LL	<0.001	621	4.678	10.5	-28.5	-43.5
GM L	R1	β_2 SCI-LL > CON-LL	0.004	550	4.415	-10.5	-42	79.5
GM R	R1	β_2 SCI-LL < CON-LL	0.009	472	5.324	40.5	-39	-46.5
GM L	R2	β_1 SCI-LL < CON-LL	0.001	401	5.615	-18	-91.5	-21
GM L	R2	β_2 SCI-LL < CON-LL	0.018	209	4.733	-18	-91.5	-19.5
GM L	R2	β_2 SCI-LL < CON-LL	0.016	217	4.440	-36	-91.5	-36
GMR	R2	β_2 SCI-UL< CON-UL	0.002	336	4.214	18	-25.5	72
GMR	R2	β_2 SCI-UL< CON-UL	0.003	325	3.999	33	-33	40.5
GMR	R2	β_2 SCI-LL < CON-LL	0.044	162	6.092	15	-34.5	-58.5
WM L	RD	β_1 SCI-UL> CON-UL	<0.001	55	>10	-21	-15	36
WM L	RD	β_1 SCI-UL> CON-UL	<0.001	37	>10	-33	-9	27

WM L	RD	β_1 SCI-UL> CON-UL	0.001	28	>10	-25.5	-6	21
WM L	RD	β_1 SCI-UL< CON-UL	<0.001	36	6.920	-7.5	-42	-37.5
WM L	RD	β_1 SCI-UL< CON-UL	<0.001	37	6.284	-6	-28.5	-31.5
WM L	RD	β_2 SCI-UL> CON-UL	0.005	20	>10	-24	-1.5	21
WM L	RD	β_2 SCI-UL> CON-UL	0.002	23	6.879	-24	-51	-42
WM L	RD	β_2 SCI-UL< CON-UL	<0.001	41	>10	-21	-15	36
WM L	RD	β_2 SCI-UL< CON-UL	0.003	22	>10	-18	-16.5	49.5
WM L	RD	β_1 SCI-LL > CON-LL	0.001	27	>10	-33	-10.5	27
WM L	RD	β_1 SCI-LL > CON-LL	<0.001	54	>10	-21	-10.5	34.5
WM L	RD	β_2 SCI-LL > CON-LL	<0.001	54	>10	-21	-15	36
WM L	RD	β_2 SCI-LL > CON-LL	0.001	25	>10	-33	-9	27
WM R	RD	β_1 SCI-UL> CON-UL	<0.001	311	>10	30	-12	36
WM R	RD	β_1 SCI-UL< CON-UL	<0.001	101	>10	19.5	-12	36
WM R	RD	β_1 SCI-UL< CON-UL	<0.001	45	>10	28.5	1.5	33
WM R	RD	β_1 SCI-UL< CON-UL	<0.001	82	6.559	10.5	-30	-36
WM R	RD	β_2 SCI-UL> CON-UL	<0.001	204	>10	30	-12	36
WM R	RD	β_2 SCI-UL> CON-UL	0.005	21	7.842	24	4.5	37.5
WM R	RD	β_2 SCI-UL< CON-UL	<0.001	103	>10	19.5	-12	36
WM R	RD	β_2 SCI-UL< CON-UL	0.002	24	>10	21	-24	40.5
WM R	RD	β_1 SCI-LL > CON-LL	<0.001	187	>10	19.5	-15	39
WM R	RD	β_1 SCI-LL < CON-LL	0.002	24	>10	21	-10.5	42
WM R	RD	β_2 SCI-LL > CON-LL	<0.001	190	>10	30	-12	36
GM L	VBM	β_1 SCI-LL < CON-LL	0.006	252	5.968	-30	-97.5	-33
GM L	VBM	β_2 SCI-LL < CON-LL	0.016	202	7.761	-9	-94.5	-46.5
GM L	VBM	β_2 SCI-LL < CON-LL	0.006	253	5.647	-21	-99	-28.5
GM R	VBM	β_1 SCI-LL > CON-LL	0.001	355	>10	24	-37.5	40.5
WM L	VBM	β_1 SCI-UL> CON-UL	0.026	71	6.590	-4.5	-28.5	-31.5
WM L	VBM	β_1 SCI-LL > CON-LL	0.007	103	>10	-27	12	21
WM L	VBM	β_1 SCI-LL > CON-LL	<0.001	223	>10	-24	-7.5	36
WM L	VBM	β_1 SCI-LL > CON-LL	0.044	59	6.140	-12	-40.5	-40.5
WM L	VBM	β_1 SCI-LL < CON-LL	<0.001	242	>10	-34.5	-9	27
WM L	VBM	β_1 SCI-LL < CON-LL	<0.001	181	>10	-4.5	-30	-33
WM L	VBM	β_1 SCI-LL < CON-LL	<0.001	280	>10	-19.5	-10.5	39
WM L	VBM	β_1 SCI-LL < CON-LL	<0.001	246	>10	-22.5	-51	-40.5
WM L	VBM	β_1 SCI-LL < CON-LL	0.042	60	>10	-22.5	-28.5	46.5
WM L	VBM	β_2 SCI-LL > CON-LL	<0.001	290	>10	-24	-7.5	36
WM L	VBM	β_2 SCI-LL > CON-LL	0.013	86	6.913	-12	-40.5	-40.5
WM L	VBM	β_2 SCI-LL < CON-LL	<0.001	221	>10	-34.5	-9	27
WM L	VBM	β_2 SCI-LL < CON-LL	0.001	170	>10	-4.5	-30	-33
WM L	VBM	β_2 SCI-LL < CON-LL	0.001	167	>10	-19.5	-10.5	39
WM L	VBM	β_2 SCI-LL < CON-LL	0.015	83	>10	-22.5	-51	-42
WM L	VBM	β_2 SCI-LL < CON-LL	0.001	149	>10	-25.5	13.5	22.5

WM R	VBM	β_2 SCI-UL> CON-UL	0.043	58	7.683	22.5	-19.5	42
WM R	VBM	β_1 SCI-LL > CON-LL	0.002	131	>10	4.5	-28.5	-37.5
WM R	VBM	β_1 SCI-LL > CON-LL	0.003	124	7.194	16.5	1.5	34.5
WM R	VBM	β_1 SCI-LL < CON-LL	<0.001	1565	>10	25.5	-13.5	40.5
WM R	VBM	β_1 SCI-LL < CON-LL	<0.001	221	>10	24	-48	-42
WM R	VBM	β_1 SCI-LL < CON-LL	0.033	64	4.858	3	-40.5	-42
WM R	VBM	β_2 SCI-LL > CON-LL	0.001	161	7.751	16.5	3	34.5
WM R	VBM	β_2 SCI-LL > CON-LL	0.031	65	5.152	3	-19.5	-36
WM R	VBM	β_2 SCI-LL > CON-LL	0.028	67	4.528	7.5	-30	-13.5
WM R	VBM	β_2 SCI-LL < CON-LL	0.004	115	7.374	24	-46.5	-40.5

Supplementary Table 6: Longitudinal statistical parametric mapping (SPM) analysis showing differences in the linear (β_1) and quadratic (β_2) time dependence of longitudinal relaxation rate (R1), magnetisation transfer saturation (MTsat), transverse relaxation rate (R2*), fractional anisotropy (FA), mean diffusivity (MD), radial diffusivity (RD), and axial diffusivity (AD) maps between spinal cord–injured (SCI) and healthy trainees, separately for upper-limb and lower-limb training groups. SCI-UL=patients upper limb trainees, SCI-LL=patients lower limb trainees, CON-UL=healthy controls upper limb trainees, CON-LL=healthy controls lower limb trainees, R = right, L = left, WM = white matter, GM = grey matter.
