# Supplementary material for: Neuronal plasticity during motor rehabilitation training after spinal cord injury
Source: Commun Biol. 2026 Mar 9;9:561. doi: 10.1038/s42003-026-09793-7 (PMC13103311; doi:10.1038/s42003-026-09793-7)
Supplement: Supplementary file 2 — Description of Additional Supplementary Files [file 42003_2026_9793_MOESM2_ESM.docx]

Description of Additional Supplementary File

File name: Supplementary Data
Description: Anonymized data underlying the graphs are provided in the Supplementary Data
