# Supplementary material for: Neuronal plasticity during motor rehabilitation training after spinal cord injury
Source: Commun Biol. 2026 Mar 9;9:561. doi: 10.1038/s42003-026-09793-7 (PMC13103311; doi:10.1038/s42003-026-09793-7)
Supplement: Supplementary file 4 — Reporting Summary [file 42003_2026_9793_MOESM4_ESM.pdf]

nature portfolio

Corresponding author(s): Prof. Patrick Freund, MD, PhD

Last updated by author(s): 20.02.2026

Reporting Summary

Nature Portfolio wishes to improve the reproducibility of the work that we publish. This form provides structure for consistency and transparency in reporting. For further information on Nature Portfolio policies, see our Editorial Policies and the Editorial Policy Checklist.

Statistics

For all statistical analyses, confirm that the following items are present in the figure legend, table legend, main text, or Methods section.

n/a	Confirmed	
☐	☒	The exact sample size (*n*) for each experimental group/condition, given as a discrete number and unit of measurement
☐	☒	A statement on whether measurements were taken from distinct samples or whether the same sample was measured repeatedly
☐	☒	The statistical test(s) used AND whether they are one- or two-sided<br>*Only common tests should be described solely by name; describe more complex techniques in the Methods section.*
☐	☒	A description of all covariates tested
☐	☒	A description of any assumptions or corrections, such as tests of normality and adjustment for multiple comparisons
☐	☒	A full description of the statistical parameters including central tendency (e.g. means) or other basic estimates (e.g. regression coefficient) AND variation (e.g. standard deviation) or associated estimates of uncertainty (e.g. confidence intervals)
☐	☒	For null hypothesis testing, the test statistic (e.g. *F*, *t*, *r*) with confidence intervals, effect sizes, degrees of freedom and *P* value noted<br>*Give P values as exact values whenever suitable.*
☐	☒	For Bayesian analysis, information on the choice of priors and Markov chain Monte Carlo settings
☐	☒	For hierarchical and complex designs, identification of the appropriate level for tests and full reporting of outcomes
☐	☒	Estimates of effect sizes (e.g. Cohen's *d*, Pearson's *r*), indicating how they were calculated

Our web collection on statistics for biologists contains articles on many of the points above.

Software and code

Policy information about availability of computer code

Data collection	All data collection and processing in this study were performed using a 3T Siemens Skyra Fit scanner (Siemens Healthineers, Erlangen, Germany; VE11C). For data proccesing SPM12 (v7487), hMRI toolbox (v0.2.0), MRtrix3, FSL version 5.0.11,and MATLAB R2016b
Data analysis	For data analysis SPM12 (v7487), SPM Anatomy Toolbox, Stata 15.0, RStudio, version 2022.07.1 and JASP, version 0.17.1 and MATLAB R2016b was used.

For manuscripts utilizing custom algorithms or software that are central to the research but not yet described in published literature, software must be made available to editors and reviewers. We strongly encourage code deposition in a community repository (e.g. GitHub). See the Nature Portfolio guidelines for submitting code & software for further information.

Data

Policy information about availability of data

All manuscripts must include a data availability statement. This statement should provide the following information, where applicable:

- Accession codes, unique identifiers, or web links for publicly available datasets
- A description of any restrictions on data availability
- For clinical datasets or third party data, please ensure that the statement adheres to our policy

Anonymized data underlying the graphs are provided in the Supplementary Data. Other anonymized data will be made available upon request from qualified investigators.

Research involving human participants, their data, or biological material

Policy information about studies with human participants or human data. See also policy information about sex, gender (identity/presentation), and sexual orientation and race, ethnicity and racism.

Reporting on sex and gender	Only participants of male sex were recruited for this study. Sex was self-reported and not independently verified. Gender identity was not assessed. Recruitment was restricted to males to minimize potential sex-related confounding effects on neuroplastic processes, which may differ in temporal dynamics, spatial distribution, or magnitude. As stated in the manuscript, this restriction may limit generalizability to females; however, the higher prevalence of traumatic spinal cord injury in men supports the relevance of the study to the majority of affected patients. Because the sample included only males, sex- or gender-based analyses could not be performed.
Reporting on race, ethnicity, or other socially relevant groupings	No socially constructed or socially relevant categorization variables (e.g., race, ethnicity, socioeconomic status, gender identity) were collected or used in this study. Participants were not classified into any such categories, and no analyses were performed based on these variables.
Population characteristics	Relevant participant characteristics included age (age ranges in 5-year groups) and clinical diagnosis. Age was recorded via self-report at enrollment. Clinical diagnosis (including neurological level and severity of spinal cord injury in the patient group) was obtained from medical records and confirmed by treating clinicians. Age was considered as a covariate in the statistical analyses where appropriate, whereas clinical diagnosis was recorded for characterization of the cohort. No genotypic information or additional treatment categories were collected.
Recruitment	Participants were recruited at the Balgrist University Hospital through clinician referral and study information provided to eligible patients and healthy volunteers. Recruitment was controlled to achieve an approximately age-matched distribution between groups. Because recruitment was limited to a single specialized center and the sample size was modest, the resulting cohort may not fully reflect the broader or more heterogeneous spinal cord injury population found in other regions or countries. These factors may introduce selection bias and may limit the generalizability of the findings, although they are unlikely to affect the internal validity of the within-study group comparisons.
Ethics oversight	The study complied with the Declaration of Helsinki and Good Clinical Practice guidelines and was approved by the Zurich Cantonal Ethics Committee (KEK-2013-0559).

Note that full information on the approval of the study protocol must also be provided in the manuscript.

Field-specific reporting

Please select the one below that is the best fit for your research. If you are not sure, read the appropriate sections before making your selection.

☒ Life sciences ☐ Behavioural & social sciences ☐ Ecological, evolutionary & environmental sciences

For a reference copy of the document with all sections, see nature.com/documents/nr-reporting-summary-flat.pdf

Life sciences study design

All studies must disclose on these points even when the disclosure is negative.

Sample size	No formal a priori sample size calculation was performed. The sample size was determined based on feasibility considerations and guided by previous neuroimaging studies in spinal cord injury that used comparable cohort sizes. Given the intensive MRI protocol and the longitudinal design, the chosen sample size reflects a balance between participant burden and practical recruitment constraints. Although the sample size is modest, it is consistent with prior work in the field and sufficient to detect robust within-group training effects, which was the primary focus of the study.
Data exclusions	Data were excluded only from the analysis assessing associations between structural and behavioral changes. Participants were removed from this specific analysis when their behavioral parameters exceeded 3 standard deviations from the group mean. This exclusion criterion was pre-established to minimize bias arising from cases in which the exponential behavioral fit was suboptimal. No other data were excluded from the study.
Replication	To ensure the reproducibility of the experimental findings, neuroplasticity-related MRI changes were first examined in a healthy control cohort using two independent quantitative MRI protocols (MPM and DTI). Consistent training-related effects were observed across these modalities, supporting the robustness of the measurements. We then investigated the SCI cohort, which demonstrated patterns of structural change comparable to those previously reported in the literature, as outlined in the manuscript. All analyses were reproducible using the described processing pipelines.
Randomization	Participants were allocated to experimental groups based on motor capacity. Patients without residual lower-limb function were assigned to the upper-limb training condition, as they could not safely perform lower-limb training. For healthy participants, the first six individuals were block-randomized to upper-limb training, lower-limb training, or a no-training control group. Thereafter, an age-matching procedure was applied to ensure comparability between healthy participants and the spinal cord injury group. Allocation was therefore partly constrained by clinical feasibility and partly optimized to control for age as a covariate.
Blinding	Investigators responsible for administering the training protocols were not blinded to group allocation, as knowledge of the assigned training condition (upper-limb, lower-limb, or control) was necessary to deliver the appropriate intervention. However, data preprocessing and

quantitative MRI analysis were performed using identical procedures across all participants. The investigator conducting the data processing was not aware of participants' behavioral performance or training outcomes until after the analyses were completed. Thus, while blinding during intervention delivery was not feasible, blinding was effectively maintained during data analysis.

Reporting for specific materials, systems and methods

We require information from authors about some types of materials, experimental systems and methods used in many studies. Here, indicate whether each material, system or method listed is relevant to your study. If you are not sure if a list item applies to your research, read the appropriate section before selecting a response.

Materials & experimental systems

n/a	Involved in the study
☒	☐ Antibodies
☒	☐ Eukaryotic cell lines
☒	☐ Palaeontology and archaeology
☒	☐ Animals and other organisms
☐	☒ Clinical data
☒	☐ Dual use research of concern
☒	☐ Plants

Methods

n/a	Involved in the study
☒	☐ ChIP-seq
☒	☐ Flow cytometry
☐	☒ MRI-based neuroimaging

Clinical data

Policy information about clinical studies

All manuscripts should comply with the ICMJE guidelines for publication of clinical research and a completed CONSORT checklist must be included with all submissions.

Clinical trial registration	*Provide the trial registration number from ClinicalTrials.gov or an equivalent agency.*
Study protocol	*Note where the full trial protocol can be accessed OR if not available, explain why.*
Data collection	Data were collected at Balgrist University Hospital between September 2014 and December 2017.
Outcomes	The pre-defined primary and secondary outcome measures were the percentage of correct stimulus responses (%CSR) and response time (RT), respectively. Behavioral improvements across training were quantified by fitting individual learning curves with an exponential model, from which the rate and magnitude of behavioral change were derived.

Plants

Seed stocks	Not applicable.
Novel plant genotypes	Not applicable.
Authentication	Not applicable.

Magnetic resonance imaging

Experimental design

Design type	longitudinal structural and quantitative MRI
Design specifications	assessments at baseline (day 0), during training (days 7, 14, 28), and at follow-up (day 84)
Behavioral performance measures	The recorded behavioral variables were the percentage of correct stimulus responses (%CSR) and response time (RT).<br>To characterize individual learning trajectories, an exponential function was fitted to each participant's performance data. This yielded three parameters:<br>-α, the asymptote (learning plateau),<br>-δ, the acquisition gain (magnitude of improvement from baseline to asymptote), and<br>-γ, the time constant (speed at which the asymptote was reached).<br>These parameters were used to verify expected task performance and quantify learning-related improvements.

Acquisition

Imaging type(s)

multi-parameter mapping (MPM) for R1, MTsat, and R2*, and diffusion MRI (dMRI) for tensor metrics (FA, MD, AD, RD).

Field strength

3T

Sequence & imaging parameters

The MPM protocol acquires three volumes using a 3Dmulti-echo spoiled gradient echo sequence (based on the Siemens FLASH product sequence) with 1mm^3 isotropic resolution and a field of view (FoV) of 240 × 256 × 176 mm^3. Each volume was acquired using a different TR and radio-frequency (RF) excitation flip angle combination to achieve images with either T1-weighting: 25 ms / 23°, proton density (PD)-weighting: 25 ms / 4°, or MT-weighting:37 ms / 9°, with an off-resonance MTsat pulse applied prior to excitation. Echoes were acquired at 6 equidistant echo times (TE) from 2.46 to 14.76 ms for all weightings, with an additional 2 echoes at 17.22 and 19.68 ms for the PD-weighted and T1-weighted volumes. Total acquisition time was 23mins.We used parallel imaging with an acceleration factor of 2 in both phase-encoding directions. Subsequent reconstruction was performed with the generalised auto-calibration partially parallel acquisition algorithm(GRAPPA) in the anterior-posterior phaseencoding direction and a partial Fourier acquisition with a 6/8 sampling factor in the partition direction left-right.

The dMRI dataset comprised 60 diffusion-weighted images with a b-value of 1200 s/mm2, each employing a unique diffusion-encoding direction, and 7 T2-weighted images with a b-value of 0 s/mm^2. These scans were obtained using a 2D single-shot spin-echo echo-planar imaging sequence that covered the entire brain. The sequence included 56 slices with a thickness of 2.5 mm and a 10% gap, acquired in an ascending interleaved order. Additional acquisition parameters were as follows: in-plane resolution of 2.5 ×2.5 mm^2, in-plane field of view measuring 220 × 220 mm^2 ,repetition time of 7600 ms, echo time of 80 ms, f lip angle of 90◦, GRAPPA (generalized auto-calibrating partially parallel acquisition) with an acceleration factor of 2 in the phase-encoding direction (anterior–posterior), 7/8 phase partial Fourier, nominal echo spacing of 0.7 ms, and readout bandwidth of 1624 Hz/pixel, for a total dMRI acquisition time of 8 min and 54 s. Additionally, a single, T2-weighted image with a b-value of 0 s/mm2, sharing the same geometry and sequence parameters but featuring an opposite phase-encoding direction (posterior–anterior), was acquired.

Area of acquisition

Whole brain

Diffusion MRI ☒ Used ☐ Not used

Parameters

See above and note that cardiac gating was not used.

Preprocessing

Preprocessing software

MPM maps were generated with the hMRI toolbox v0.2.0 in SPM12 (v7487) and Matlab 2016b, using UNICORT to correct for transmit field inhomogeneities. All MPM maps in MNI space were spatially smoothed using a Gaussian kernel with 5-mm (in GM) and 3-mm (in WM) full-width at half-maximum (FWHM) while appropriate tissue segment weighting was used to minimise partial volume effects of GM/WM (Draganski et al.,2011). All DTI maps, in MNI space were spatially smoothed using a tissue-specific 5 mm full-width at half-maximum Gaussian kernel within both the GM and WM (Draganski et al.,2011).

Normalization

MTsat maps were first skull-stripped. For skull-stripping, the MTsat maps were segmented using the "Segment Longitudinal Data" of the CAT12 toolbox (CAT12.6 (r1450 with the graph-cut/region-growing (GCUT) approach. After this skull-skipping step, the MTsat maps were used for longitudinal registration within-participants, based on a generative model, in which each image volume was registered to a subject-specific average map, combining non-linear and rigid-body registration with corrections for intensity bias artefacts (Ashburner,2013). This procedure generated participant-specific, mid-point maps with corresponding deformation fields. Second, a unified segmentation was applied to the subject's midpoint map, generating probability maps of GM, WM, and cerebrospinal fluid (CSF). Third, nonlinear template generation and image registration was applied to subject-specific, midpoint GM and WM tissue maps based on Dartel (Ashburner,2007) and the resulting template was registered to Montreal Neurological Institute (MNI) space using an affine transform. Fourth, all MPM and co-registered DTI maps were warped to MNI space using the transformations obtained in the previous steps.

Normalization template

MNI152 space

Noise and artifact removal

For denoising the diffusion data, MRtrix3 was used.

Volume censoring

No MRI volumes were censored. The only exclusions applied in this study concerned behavioral outliers, which were removed as described above.

Statistical modeling & inference

Model type and settings

The model for the training induced brain changes and topological changes incorporated an intercept and a linear and quadratic time effect for each subject separately. The general linear model included predictors (intercept, time,time^2) and covariates (age, total intracranial volume [TIV]). The time and time^2 parameters were mean-centred and the linear component was orthogonalized with respect to the quadratic component, enabling the identification of both linear and transient negative equadratic (low-high-low) and transient positive quadratic (high-low-high) trajectories. To analyse the association between MRI and motor learning parameters, we used SPM's multiple linear regression models to test for associations between linear and quadratic structural changes (linear or quadratic beta from each subject) with behavioural improvement (?), and speed of improvement(?), for each participant-specific model, using both training parameters (%CSR

and RT). For this association analysis, we have excluded parameters exceeding the three standard deviations from the group mean.

Effect(s) tested

A full factorial design was implemented in SPM with each subject entered as a separate level. For each subject, all available longitudinal MRI scans were included in the model. Time and time² were entered as orthogonalized covariates to characterize linear and non-linear longitudinal effects, whereas follow-up timepoints were excluded from the model. This design treats subjects as independent random effects and allows for unequal variance across subjects. Age and total intracranial volume (TIV) were included as covariates of no interest.

Specify type of analysis: ☐ Whole brain ☐ ROI-based ☒ Both

Anatomical location(s)

Whole WM and GM were tested, as well as combined anatomical regions for each hemisphere, including the sensorimotor cortices (M1), cranial corticospinal tract (CST), thalamus, cerebellum, and the hippocampal formation comprising the entorhinal cortex (EC), hippocampus, dentate gyrus, cornu ammonis, and subiculum.

Statistic type for inference

(See Eklund et al. 2016)

Cluster-wise statistical inference was performed. Significant clusters were identified using a conservative cluster-forming threshold of $p = 0.001$ (uncorrected). All results were corrected for multiple comparisons using family-wise error (FWE) correction at $p < 0.05$ (one-sided), and clusters comprising ≥20 voxels were considered significant.

Correction

Family-wise error

Models & analysis

n/a	Involved in the study
☒	☐ Functional and/or effective connectivity
☒	☐ Graph analysis
☒	☐ Multivariate modeling or predictive analysis
